# A cross-sectional analysis of psychosocial and structural barriers and facilitators associated with PrEP use among a sample of transgender women in Chicago, IL

**DOI:** 10.1186/s12981-023-00516-0

**Published:** 2023-04-21

**Authors:** Lisa M. Kuhns, Judy Perloff, Amy K. Johnson, Josie Lynne Paul, Kevin Pleasant, Kaiji Evans, Damian J. Denson, Deborah J. Gelaude, Patricia A. Bessler, Rose Diskin, Marbella Cervantes, Robert Garofalo, Anna L. Hotton

**Affiliations:** 1 Department of Pediatrics, Northwestern University, Feinberg School of Medicine, Chicago, IL USA; 2 Division of Adolescent and Young Adult Medicine, Ann & Robert H. Lurie Children’s Hospital of Chicago, Chicago, IL USA; 3 Chicago House and Social Service Agency, Chicago, IL USA; 4 Centers for Disease Control and Prevention, Atlanta, GA USA; 5 Department of Medicine, University of Chicago, Chicago, IL USA

**Keywords:** HIV, Transgender women, Pre-exposure prophylaxis

## Abstract

**Background:**

Expanding pre-exposure prophylaxis (PrEP) among transgender women in the United States is an important strategy to meet national HIV prevention goals, however self-reported use of PrEP is low in this group.

**Methods:**

This study reports the findings of a cross-sectional analysis of the relationship of barriers as well as facilitators to recent PrEP use among transgender women enrolled in an evaluation of the TransLife Care project (Chicago, Illinois), a structural intervention designed to meet basic needs. We computed multivariable prevalence ratios for barriers, facilitators and recent PrEP use, controlling for demographics.

**Results:**

Findings suggest that psychosocial and structural barriers, including moderate/high alcohol use, stimulant use, and history of incarceration were all positively associated with recent PrEP use among urban transgender women. In addition, a psychosocial facilitator, gender affirmation, was positively associated with recent PrEP use, while, while collective self-esteem, a was negatively associated with it. Finally, common indications for PrEP have high sensitivity, but low specificity and predictive value for identifying those on PrEP.

**Conclusion:**

We conclude that despite a large gap in PrEP use among those with indications, individuals experiencing psychosocial and structural barriers are more likely to use PrEP, and facilitators, such as psychological sense of affirmed gender may support its use.

**Trial registration:**

N/A.

## Background

Transgender (trans) women in the United States (U.S.) disproportionately acquire HIV, with an estimated HIV prevalence of 17–62% (varying by race/ethnicity) in a recent 7-city Centers for Disease Control and Prevention (CDC) study [1]. The Ending the HIV Epidemic in the U.S. initiative aims to address these disproportionate rates and reduce new HIV infections by 90% by 2030, [2] in part by intensifying HIV prevention efforts and expanding evidence-based and scalable HIV prevention approaches, including pre-exposure prophylaxis (PrEP), to highly impacted sub-groups. However, while PrEP awareness among trans women was high (92%), self-reported recent PrEP use (prior 12 months) was low (32%) [1]. Perhaps more crucially, only a small percentage of trans women with indications for PrEP are taking it [3–5], suggesting gaps in knowledge and the receipt of PrEP among those who may benefit the most. While the Centers for Disease Control and Prevention Clinical Practice Guideline for PrEP [6] specifies that all sexually active patients should be told about PrEP and it should be prescribed to anyone who asks for it, taking a sexual history and providing patient-specific risk-reduction services, depending on HIV acquisition risk, is also recommended. In this context, specific indications for PrEP include: having a sexual partner living with HIV with unknown or detectable viral load, having condomless sex with partners of unknown HIV status, or recent diagnosis with a sexually transmitted infection. Barriers to PrEP use among trans women include psychosocial factors, including mental health and substance use issues, prior negative experiences in healthcare settings, and concerns about PrEP interactions with hormone therapy; as well as structural barriers including employment and housing insecurity [7, 8]. Few studies have assessed facilitators of PrEP uptake among trans women, although it has been argued that gender-affirming providers and supportive clinic environments are essential to promote PrEP uptake [9, 10]. In addition, psychological gender affirmation reflects an internal sense of comfort and satisfaction with one’s own gender identity, body, and gender expression [11], which may support protective health behaviors. In this study, in addition to barriers, we sought to assess potential facilitators of PrEP use including structural factors such as stable housing, employment and access to insurance, as well as gender affirmation and collective self-esteem. Finally, we aimed to determine the degree to which various PrEP indications, alone and in combination, corresponded to PrEP use among trans women. Among those reporting PrEP use, we examined the proportion with indications for PrEP (i.e., sensitivity), and among those reporting one or more PrEP indications, the proportion currently using PrEP (i.e., positive predictive value).

## Methods

### Study sample

Between January 2019 and February 2020, 99 transgender women were enrolled using a convenience sampling approach, in an evaluation of the TransLife Care (TLC) project at Chicago House and Social Service Agency in Chicago, Illinois. The aim of the evaluation was to assess the efficacy of TLC to reduce risk for HIV acquisition. TLC was designed to meet the needs of racially/ethnically diverse urban transgender women by addressing the psychosocial and structural drivers of HIV, including housing, employment, legal aid and health services [12]. Eligibility criteria included: (a) identifying as transgender, transfeminine, and/or female and assigned male sex at birth; (b) ≥ 17 years of age; (c) self-reported history of sex with men in the past 4 months; (d) negative/non-reactive HIV screening test at baseline; (e) able to speak/understand English; (f) willing and able to provide informed consent; (g) intend to reside in the local area throughout the 8-month follow-up period; and (h) had no exposure to any component of the TLC intervention in the prior 4 months. Individuals were excluded if they were unable to provide informed consent due to severe mental or physical illness, or intoxication at the time of interview (those excluded could re-screen if symptoms resolved).

Described herein is a cross-sectional analysis of data collected at the baseline enrollment visit. All study procedures were approved by the Institutional Review Board of Ann & Robert H. Lurie Children’s Hospital of Chicago with a waiver of parental permission for participants aged 17 and written consent obtained for all participants.

### Data collection and measures

At the point of enrollment, participants completed a baseline questionnaire via computer-assisted interviewing, which included demographic characteristics, health behaviors, and psychosocial factors. Data for the enrollment visit were collected on-site at the TLC. Participants received $50 token of appreciation for completion of the baseline assessment.

### Demographics

Sociodemographic information collected included age (in years), race/ethnicity, sexual orientation/identity, highest level of education, and current income.

### Barriers

Structural factors. We assessed recent homelessness with a two-part question: “In your lifetime, have you ever been homeless at all? That is, you slept in a shelter for homeless people, on the streets, at a friend or relative’s house for a few nights or weeks, or another place not intended for sleeping?” (Yes, No); “In the past 4 months, were you homeless at any time?” (Yes, No). History of arrest and incarceration were assessed each with a single question: “Have you ever been arrested by the police?” (Yes, No); “Have you ever been in jail, prison, police lock-up, immigrant detention or juvenile detention?” (Yes, No).

Psychosocial factors. We assessed depression and anxiety symptoms with the 10-item version of the Center for Epidemiological Studies Depression Scale (CESD-10) [13] and the 7-item Generalized Anxiety Disorder (GAD-7) [14], respectively, and exposure to transgender-specific victimization with a 10-item victimization scale [15], adapted for trans women. We assessed substance use with the World Health Organization Alcohol, Smoking, and Substance Involvement Screening Test (ASSIST), which includes assessment of 10 substances and related problems. Following coding guidelines for the CESD-10, GAD-7 and ASSIST, respectively, participants were coded as having high versus low depressive symptoms (CESD-10; ≥10, < 10), minimal, mild, moderate or severe anxiety symptoms (GAD-7; 0–4, 5–9, 10–14, 15–21), and low, medium or high substance use (alcohol: 0–10, 11–26, 27–36; cannabis, stimulants, opioid/sedatives: 0–3, 4–26, 27–36) for each substance.

### Facilitators

Structural factors. We assessed health insurance status with a single question (“What kind of insurance do you currently use to pay for health care?” Responses were coded as: any insurance (including Medicaid/Medicare, private, other) versus none. We assessed stable housing with a single item, “Which of the following best describes your current living situation? By current living situation, we mean where have you been staying during the past seven days?” Responses coded to reflect “your own place, a room, apartment, or house that is your home” versus all others (e.g., jail/prison, drug treatment, transitional housing, hotel/motel). We assessed current employment with the item, “Please indicate which of the following is true for you regarding your current work status? The responses were coded with “working for pay at a job or business,” versus all others.

Psychosocial factors. We measured psychological gender affirmation using a 5-item measure of comfort and satisfaction with affirmation on a 5-point scale (e.g., “How comfortable are you with people knowing that you are transgender? Not at all comfortable, slightly comfortable, moderately comfortable, very comfortable, extremely comfortable) [17]. We measured transgender-specific collective self-esteem (CSES) with a 16-item measure of thoughts and feelings related to being part of the transgender community on a 7-point agreement scale (i.e., strongly disagree to strongly agree) with four sub-scales: membership (how “good” or “worthy” they feel as a member of the group), private (how good they feel about their group), public (how others are perceived to view the group) and identity (how important the group is to their self-concept) [18].

### PrEP indications

We measured recent (past 30 days) condomless anal/vaginal sex (Yes, No) and recent history of HIV-positive sexual partners with items from the AIDS Risk Behavior Assessment (ARBA), adapted for trans women [16]. We measured exchange sex with two sequential items referencing anal and vaginal sex respectively: “How many partners have you had anal (insertive or receptive)/vaginal sex with in the past month? This includes sex with or without a condom?” “How many did you have anal/vaginal sex with, in exchange for things you needed (like money, drugs, food, shelter, etc.)?” Responses were coded to reflect any exchange sex (anal/vaginal) in the past month. We measured history of sexually transmitted infections (STIs) with a single question: “Have you ever been told by a doctor or nurse that you had a sexually transmitted infection, other than HIV?” (Yes, No).

### PrEP use

We measured ever having used PrEP, used PrEP in the last four months, and used PrEP in the last month as well as adherence to PrEP with a series of items used in a prior study of trans women [3]: “Have you ever taken HIV medication before sex because you thought it would lower your chances of getting HIV (also known as PrEP)?” (Yes, No); “Have you taken PrEP in the last 4 months?” “Have you taken PrEP in the last month?” “Please indicate whether or not you have taken PrEP on each day during the past month, beginning with yesterday (timeline follow-back approach with calendar; coded on a scale of 0-100% adherent). For analysis, we created binary variables for PrEP use in the recent four months and in the past one month, respectively, versus none/not recent, which included those who had never used PrEP and those who had used PrEP previously, (i.e., > 4 months or > 1 month ago, but not recently). We also created a 3-category variable including never used, past use (having ever taken PrEP but not in the past four months), and recent use (having taken PrEP in the past four months). We present the results descriptively using all three PrEP recall variables. We used the binary recent four month use for multivariable analysis because the small sample size limited our ability to conduct multinomial analysis. For analysis of predictive accuracy, we used past one month PrEP use as the outcome because it corresponded most closely with the assessment period of the PrEP indications.

### Analysis

To assess bivariate associations between barriers and facilitators and recent PrEP use, we calculated chi-square statistics for categorical variables and Kruskal-Wallis tests for continuous variables. We used multivariable Poisson regression with robust error variance to compute univariable and multivariable prevalence ratios for associations between barriers and facilitators and recent 4-month PrEP use, controlling for age, race/ethnicity, insurance, and income. To assess the accuracy of PrEP indications alone and in combination for identifying PrEP users, we computed sensitivity (i.e. the proportion of those on PrEP with any indication for PrEP), specificity (i.e., the proportion of those not on PrEP without an indication for PrEP), positive predictive value (i.e., the proportion taking PrEP among those with PrEP indications), and area under the receiver operating characteristic (ROC) curve (an overall measure of predictive accuracy, where 0.5 indicates no better than chance and higher values indicate higher predictive performance). All analyses were conducted in Stata version 17.0 (Stata Corp, College Station, TX).

## Results

Participants were aged 27.7 years (SD = 9.3) on average, with the majority, 81.9%, identifying as Black or African American non-Hispanic, Other non-Hispanic or Hispanic in terms of race/ethnicity (Table 1). Most, 67.0%, reported a high school education/GED or less. Structural barriers were common, with a history of arrest/incarceration and homelessness in the last four months reported by 45.7% and 28.7% of the sample respectively. A total of 16.0% reported recent 4-month use. Among those reporting recent PrEP use (4-month), 12 (of 15) used PrEP within the last 28 days, and 75% of that group reported 100% adherence.

**Table 1:** Bivariate comparison of characteristics of transgender women enrolled in the TLC study by recent, past and no PrEP use

	Total, n (col %) or M (SD)	Recent use (within 4 months) n (row%) or M (SD)	Past use (> 4 months ago) n (row%) or M (SD)	Never used n (row%) or M (SD)	p-value^a^
Total	94 (100.0)	15 (16.0)	15 (16.0)	64 (68.1)	--
Age, M (SD)	27.7 (9.3)	28.9 (8.5)	25.6 (10.1)	27.9 (9.4)	0.139
Race/ethnicity					
White NH	17 (18.1)	5 (29.4)	0 (0.0)	12 (70.6)	0.260
Black NH	52 (55.3)	7 (13.5)	12 (23.1)	33 (63.5)	
Latin/Hispanic	19 (20.2)	2 (10.5)	2 (10.5)	15 (79.0)	
Other NH	6 (6.4)	1 (16.7)	1 (16.7)	4 (66.7)	
Sexual identity					
Gay or lesbian	22 (23.4)	2 (9.1)	6 (27.3)	14 (63.6)	0.537
Straight	30 (31.9)	4 (13.3)	4 (13.3)	22 (73.3)	
Bisexual	14 (14.9)	4 (28.6)	1 (7.1)	9 (64.3)	
Other/unknown	28 (19.8)	5 (17.9)	4 (14.3)	19 (67.9)	
Education					
Less than HS	25 (26.6)	6 (24.0)	4 (16.0)	15 (60.0)	0.156
HS or GED	38 (40.4)	6 (15.8)	9 (23.7)	23 (60.5)	
More than HS	31 (33.0)	3 (9.7)	2 (6.5)	26 (83.9)	
Income					
<10k	65 (69.2)	7 (10.8)	11 (16.9)	47 (72.3)	0.249
10-20k	10 (10.6)	2 (20.0)	2 (20.0)	6 (60.0)	
≥20k	19 (20.2)	4 (28.6)	0 (0.0)	10 (71.4)	
Ever arrested/incarcerated					
No	51 (54.3)	4 (8.0)	5 (10.0)	41 (82.0)	0.008
Yes	43 (45.7)	11 (25.0)	10 (22.7)	23 (52.3)	
Insurance					
None	36 (38.3)	3 (8.3)	4 (11.1)	29 (80.6)	0.076
Medicare/Medicaid	39 (41.5)	7 (18.0)	10 (25.6)	22 (56.4)	
Private/other	19 (20.2)	5 (26.3)	1 (5.3)	13 (68.4)	
Homeless, past 4 m					
No	67 (71.3)	9 (13.4)	8 (11.9)	50 (74.6)	0.093
Yes	27 (28.7)	6 (22.2)	7 (25.9)	14 (51.9)	
100% PrEP adherence in past 28d (n = 12)	--	9 (75.0)	--	--	--
***Psychosocial variables***					
CESD-10					
<10	56 (59.6)	5 (8.9)	10 (17.9)	41 (73.2)	0.077
≥10	38 (40.4)	10 (26.3)	5 (13.2)	23 (60.5)	
GAD-7					
Mild	34 (36.2)	4 (11.8)	4 (11.8)	26 (76.5)	0.775
Moderate	23 (34.5)	4 (17.4)	4 (17.4)	15 (65.2)	
Severe	37 (39.4)	7 (18.9)	7 (18.9)	23 (62.2)	
Victimization scale, M (SD)	1.8 (1.6)	1.8 (0.7)	2.5 (2.7)	1.6 (1.4)	0.004
Alcohol use					
Low	80 (85.1)	10 (12.5)	13 (16.3)	57 (71.3)	0.089
High/Moderate	14 (14.9)	5 (35.7)	2 (14.3)	7 (50.0)	
Cannabis use					
Low	46 (48.9)	4 (8.7)	3 (6.5)	39 (84.8)	0.003
High/Moderate	48 (51.1)	11 (22.9)	12 (25.0)	25 (52.1)	
Stimulant use					
Low	80 (85.1)	8 (10.0)	11 (13.8)	61 (76.3)	< 0.001
High/Moderate	14 (14.9)	7 (50.0)	4 (28.6)	3 (21.4)	
Opioid/sedative use					
Low	88 (93.6)	12 (13.6)	14 (15.9)	62 (70.5)	0.055
High/Moderate	6 (6.4)	3 (50.0)	1 (16.7)	2 (33.3)	
*Protective factors*					
Stably housed currently					
No	44 (47.3)	5 (11.1)	9 (20.0)	31 (68.9)	0.339
Yes	49 (52.7)	10 (20.4)	6 (12.2)	33 (67.4)	
Currently employed					
No	80 (85.1)	14 (17.5)	13 (16.3)	53 (66.3)	0.578
Yes	14 (14.9)	1 (7.1)	2 (14.3)	11 (78.6)	
Gender affirmation, M (SD)		3.8 (0.6)	3.4 (0.6)	3.5 (0.9)	0.247
Collective self esteem					
Identity, M (SD)	5.3 (1.1)	5.0 (1.1)	5.3 (1.1)	5.4 (1.1)	0.485
Membership, M (SD)	5.4 (1.3)	4.9 (1.3)	5.5 (1.2)	5.6 (1.3)	0.157
Private, M (SD)	4.3 (1.2)	4.1 (1.3)	4.3 (0.9)	4.4 (1.2)	0.595
Public, M (SD)	4.5 (1.3)	4.5 (1.3)	4.3 (1.1)	4.6 (1.3)	0.533
Social support, M (SD)	2.9 (1.2)	2.5 (1.4)	3.1 (1.2)	2.9 (1.2)	0.343
***Behavioral PrEP indications***					
STI history (ever)					
No	69 (73.4)	8 (11.6)	5 (7.3)	56 (81.2)	< 0.001
Yes	25 (26.6)	7 (28.0)	10 (40.0)	8 (32.0)	
Condomless vaginal/anal sex, past 1 m					
No	48 (51.1)	6 (12.5)	8 (16.7)	34 (70.8)	0.646
Yes	46 (48.9)	9 (19.6)	7 (15.2)	30 (65.2)	
Any HIV positive partners past 1 m					
No	87 (92.5)	12 (13.8)	12 (13.8)	63 (72.4)	0.006
Yes	7 (7.5)	3 (42.9)	3 (42.9)	1 (14.3)	
Exchange sex past 1 m					
No	69 (73.4)	8 (11.6)	4 (5.8)	57 (82.6)	< 0.001
Yes	25 (26.6)	7 (28.0)	11 (44.0)	7 (28.0)	

a. P-values by Pearson chi-square test or Kruskal-Wallis test for overall differences across groups

In bivariable analysis (Table 1), comparing individuals who never used PrEP to past and recent PrEP users, differences were significant for several of the structural and psychosocial barriers, including history of arrest and/or incarceration, victimization, cannabis and stimulant use, and for indications for PrEP, including history of STIs, having an HIV-positive sex partner and a history of exchange sex.

**Table 2:** Multivariable regression: factors associated with PrEP use in the recent 4 months among transgender women participating in the TLC study (n = 94)

	PR (95% CI)	p-value	aPR^a^ (95% CI)	p-value
***Barriers***				
Homeless in past 4 m	1.65 (0.65–4.22)	0.292	1.59 (0.66–3.79)	0.299
Ever arrested or incarcerated	3.13 (1.07–9.17)	0.038	2.91 (1.03–8.20)	0.043
CESD-10 ≥ 10 vs. <10	2.95 (1.09–7.99)	0.034	2.73 (0.96–7.78)	0.060
GAD-7				
Moderate	1.48 (0.41–5.36)	0.552	1.05 (0.29–3.77)	0.946
High	1.61 (0.51–5.04)	0.415	1.03 (0.31–3.41)	0.956
Alcohol use^b^	2.86 (1.14–7.14)	0.025	2.80 (1.05–7.50)	0.040
Cannabis use^b^	2.64 (0.90–7.73)	0.078	2.93 (0.97–8.91)	0.057
Stimulant use^b^	5.00 (2.15–11.6)	< 0.001	3.97 (1.47–10.8)	0.007
Nonmedical opioid use^b^	3.67 (1.40–9.60)	0.008	3.80 (0.94–15.4)	0.061
Victimization scale	1.01 (0.85–1.20)	0.893	0.96 (0.78–1.17)	0.672
***Facilitators***				
Currently stably housed	1.80 (0.66–4.88)	0.251	1.38 (0.50–3.78)	0.532
Currently employed	0.41 (0.06–2.89)	0.370	0.22 (0.03–1.48)	0.120
Gender affirmation	1.50 (0.98–2.29)	0.060	1.92 (1.04–3.54)	0.037
Collective self esteem				
Identity	0.81 (0.56–1.18)	0.281	0.74 (0.49–1.12)	0.151
Membership	0.76 (0.57–1.01)	0.055	0.71 (0.51-1.00)	0.052
Private	0.82 (0.56–1.21)	0.320	0.81 (0.50–1.30)	0.378
Public	0.98 (0.69–1.39)	0.911	0.96 (0.73–1.27)	0.787
Social support	0.76 (0.49–1.18)	0.216	0.63 (0.36–1.09)	0.100
***PrEP indications***				
STI history (ever)	2.42 (0.97-6.00)	0.058	2.70 (1.09–6.65)	0.031
Any condomless vaginal/anal sex (past 1 m)	1.57 (0.60–4.07)	0.358	1.35 (0.54–3.36)	0.521
Any HIV positive partners (past 1 m)	3.11 (1.13–8.52)	0.028	2.40 (0.78–7.37)	0.125
Exchange sex (past 1 m)	2.42 (0.97-6.00)	0.058	2.05 (0.83–5.11)	0.121

a. Estimates were adjusted for age, race/ethnicity, insurance status, and income; aPR = adjusted prevalence ratio

b. Substance use was measured using the ASSIST scale, dichotomized as high/moderate vs. low

In multivariable analysis of recent 4-month PrEP use (Table 2), controlling for age, race/ethnicity insurance status, and income, several barriers were statistically significant, including high/moderate alcohol (aPR = 2.80, 95% CI, 1.05–7.50) and stimulant use (aPR = 3.97, 95% CI, 1.47–10.8), and history of arrest or incarceration (aPR = 2.91, 95% CI = 1.03–8.20). In terms of protective factors, gender affirmation was positively associated with recent PrEP use (aPR = 1.92, 95% CI, 1.04–3.54) and the membership subscale of collective self-esteem was negatively associated with it (aPR = 0.71, 95% CI, 0.51-1.00). Finally, an indication for PrEP, history of STIs, was also associated with PrEP use (aPR = 2.70, 95% CI, 1.09–6.65).

In terms of predictive accuracy of PrEP indications for identifying those with very recent PrEP use (past one month), the combined set of indications (condomless sex, HIV-positive partner and STI history) had high sensitivity, at 91.7% (95% CI, 61.5-99.8%), meaning that most people taking PrEP had at least one indication for PrEP (Table 3). Adding an indication for history of exchange sex did not increase the sensitivity of the index. In terms of specificity, the combined set of indications had low specificity to correctly identify those not on PrEP at 41.5% (95% CI, 30.7-52.9%). Having an HIV-positive partner had the best specificity, 93.9% (95% CI, 86.3-98.0%), followed by STI history at 75.6% (95% CI, 64.9-84.4%) and condomless sex at 52.4% (95% CI, 41.1-63.6%). Again, adding exchange sex did not improve specificity of the set of indications, although as a stand-alone indication, it had greater specificity at 75.6% (95% CI, 64.9-84.4%) than condomless sex and the same specificity as STI history. In terms of PPV, the probability that those indicated for PrEP were taking PrEP was low at 18.6% (95% CI, 9.7-30.9%) for the set of 3 indications and was not increased with the addition of exchange sex. The area under the ROC for the set of three indications was 0.67 (0.57–0.76), indicating relatively low predictive accuracy for distinguishing those on PrEP from those not on PrEP, again not improved with the addition of exchange sex.

**Table 3:** Sensitivity, specificity, and positive predictive value of indications for PrEP use in the past month among a sample of transgender women in the Chicago area

Past month indications for PrEP	Sensitivity^a^ % (95% CI)	Specificity^b^ % (95% CI)	1-Specificity^c^ % (95% CI)	PPV^d^ % (95% CI)	Area under the ROC curve 95% CI
Condomless vaginal/anal sex	58.3 (27.7–84.8)	52.4 (41.1–63.6)	47.6 (36.9–58.5)	15.2 (6.3–28.9)	0.55 (0.40–0.71)
Any HIV positive partners	16.7 (2.1–48.4)	93.9 (86.3–98.0)	6.1 (2.5–14.0)	28.6 (3.7–71.0)	0.55 (0.44–0.67)
STI history (ever)	41.7 (15.2–72.3)	75.6 (64.9–84.4)	24.4 (16.2–35.0)	20.0 (6.8–40.7)	0.59 (0.43–0.74)
Exchange sex	41.7 (15.2–72.3)	75.6 (64.9–84.4)	24.4 (16.2–35.0)	20.0 (6.8–40.7)	0.59 (0.43–0.74)
Combined index^e^	91.7 (61.5–99.8)	41.5 (30.7–52.9)	58.5 (47.5–68.8)	18.6 (9.7–30.9)	0.67 (0.57–0.76)
Combined index^e^ + exchange sex	91.7 (61.5–99.8)	39.0 (28.4–50.4)	61.0 (49.4–71.0)	18.0 (9.4–30.0)	0.65 (0.56–0.75)

Abbreviations: PPV, positive predictive value; ROC, receiver operating characteristic

a. Proportion with PrEP indication among those on PrEP

b. Proportion without PrEP indication among those not on PrEP

c. Proportion with PrEP indication among those not on PrEP

d. Proportion on PrEP among those with PrEP indication

e. Combined index = CAS, HIV pos, STI history

## Discussion

Findings of this study suggest that both barriers (i.e., moderate/high alcohol use, stimulant use, history of incarceration) and facilitating factors (gender affirmation, collective self-esteem), as well as history of STIs, an indication for PrEP, are associated with recent PrEP use among urban trans women vulnerable to HIV acquisition. In addition, we found that common indications for PrEP (as a set), including condomless sex, having an HIV-positive partner and history of STIs, have high sensitivity, but low specificity and predictive value for identifying those on PrEP. In other words, while most of those on PrEP had some indications, many of those not on PrEP also reported indications for PrEP, and few of those with indications for PrEP were actually using PrEP.

PrEP use in the past 12 months was reported by only 32% of transgender women in a recent CDC study, [1] and in this study, we also found low rates of recent PrEP use: 16% of trans women in our study reported use of PrEP in the last four months (based on self-report). Limited findings herein suggest that among those taking PrEP very recently (last month), adherence was high. Similarly in a prior study among trans women taking PrEP, there was evidence of high adherence, but also intermittent stoppage over time [19].

In terms of barriers, the finding that high/moderate alcohol and stimulant use are associated with recent PrEP use suggests a degree of public health success given the common association of substance use, particularly stimulant use, with HIV acquisition. Similarly, a key indication of HIV acquisition, history of STIs was associated with recent PrEP use, in multivariable analysis which also suggest more uptake among those most vulnerable, on average.

While we did not find that stable housing and employment were associated with recent PrEP use, we did find that gender affirmation was positively associated with it. In a prior studies, gender non-affirmation has been identified as a barrier to PrEP uptake [8] and was associated with lower PrEP use in sexually active trans women and men [20]. Psychological gender affirmation, which reflects an internal sense of comfort and satisfaction with one’s own gender identity, body, and gender expression, and which has been associated with resilience, positive affect, and lower depression, may serve to protect against transgender related stress and promote protective health behaviors [11]. This sample was comprised primarily of women of color and evidence suggests that psychosocial gender affirmation, in particular, is associated with positive mental health and resiliency in urban Black trans women [21]. However, we were surprised to find that the membership subscale of collective self-esteem was negatively associated with PrEP use. Given a prior study in which lower scores for this same scale were associated with indications for PrEP [3], those with lower collective self-esteem may be more likely to be on PrEP or alternatively, higher self-esteem may be related to lower perceived likelihood of HIV acquisition.

Our finding regarding the specificity and sensitivity of indications for PrEP suggest that while sensitivity is high, specificity is low. Putting these two findings together, the probability (PPV) is only 18.6% that those with indications for PrEP are actually taking it. This represents a very large gap and highlights the need for public health resources and attention to address it.

In terms of limitations, the TLC is a drop-in center that serves transgender individuals seeking assistance with basic health and social service needs and those who enrolled in the evaluation may have been different from all program participants; therefore, these findings may not be generalizable to the larger population of urban trans women. As well, this sample was comprised primarily of women of color, who may experience barriers related to race and ethnicity, which were unmeasured in this study. Similarly, this was a highly vulnerable sample of trans women, as evidenced by high exposure to structural barriers and high levels of psychosocial conditions, which may have restricted the range of our protective factors, potentially contributing to low power to detect related associations with PrEP use. However, this sample may have been particularly well-suited for the analysis of sensitivity and specificity given their relatively high prevalence of PrEP indications. As well, this was a cross-sectional analysis and thus we were not able to establish the temporal sequence between PrEP indications and PrEP use and measures of PrEP uptake. PrEP uptake and adherence were self-reported, which may be subject to recall bias. In addition, we did not measure whether participants were offered PrEP and elected not to take it, which has implications for interventions to promote PrEP uptake. The small sample size and relatively low outcome prevalence may have contributed to a lack of precision in the point estimates and insufficient power to control for all potential confounders. Finally, in terms of our use of STI history as an indication for PrEP, we only measured lifetime, rather than recent STI history, so this may have attenuated the association of this indication with recent PrEP use.

## Conclusions

We conclude that despite overall low rates of PrEP use among urban trans women in this sample, and a large gap in use among those with indications for PrEP, individuals experiencing psychosocial and structural barriers (compared to those not reporting barriers) are more likely to use PrEP, and facilitators, such as gender affirmation may support its use.

## Data Availability

The data are available upon request to and approval by the project multiple principal investigators (lkuhns@luriechildrens.org; jperloff@chicagohouse.org).

## List of Abbreviations

aPR: Adjusted Prevalence Ratio.
ARBA: AIDS Risk Behavior Assessment.
ASSIST: Alcohol, Smoking, and Substance Involvement Screening Test.
CDC: Centers for Disease Control and Prevention.
CESD-10: Center for Epidemiological Studies Depression Scale (10-item).
CSES: Collective Self-Esteem.
GAD-7: General Anxiety Disorder (7-item).
GED: General Educational Development test.
PrEP: Pre-exposure Prophylaxis.
ROC: Receiver Operating Characteristic.
STI: Sexually Transmitted Infection.
TLC: Trans Life Care.
US: United States.
